# Potential antiviral agents of *Rosmarinus officinalis* extract against herpes viruses 1 and 2

**DOI:** 10.1042/BSR20200992

**Published:** 2020-06-10

**Authors:** Wafa A. AL-Megrin, Norah A. AlSadhan, Dina M. Metwally, Razan A. Al-Talhi, Manal F. El-Khadragy, Lina J. M. Abdel-Hafez

**Affiliations:** 1 Biology Department, Faculty of Science, Princess Nourah Bint Abdulrahman University, Riyadh, Kingdom of Saudi Arabia; 2 College of Medicine, Almaarefa University, Riyadh, Kingdom of Saudi Arabia; 3 Department of Zoology, Faculty of Science, Faculty of Science, King Saud University, Riyadh, Kingdom of Saudi Arabia; 4 Parasitology Department, Faculty of Veterinary Medicine, Zagazig University, Zagazig, Egypt; 5 Department of Zoology and Entomology, Faculty of Science, Helwan University, Cairo, Egypt; 6 Department of Microbiology and Immunology, Faculty of Pharmacy, October 6 University, Giza, Egypt

**Keywords:** Antioxidant, Antiviral, Herpes simplex virus, Natural product, Rosmarinus officinallis L

## Abstract

Herpes simplex viruses 1 and 2 (HSV-1 and HSV-2) belong to the herpesviridae family and cause neurological disorders by infecting the nervous system. The present study aimed to investigate the effects of *Rosmarinus officinalis* L. (rosemary) extract against HSV-1 and HSV-2 *in vitro*. The antioxidant activity of this extract was investigated by superoxide anion and 2,2-diphenyl-1-picrylhydrazyl (DPPH) free-radical assays. Rosemary extract was evaluated by an HSV-1 antiviral assay, in which viral replication in Vero cells was determined and quantified using a cytopathic effect assay. The present study showed that rosemary extract at 30 µg/ml caused 55% inhibition of HSV-1 plaques, whereas 40 µg/ml rosemary extract caused 65% inhibition of HSV-2 plaques. The extracts completely inhibited HSV-1 and HSV-2 plaque formation at 50 µg/ml. Scavenging activity of the superoxide anion radical was observed at 65.74 mg/ml, whereas 50% scavenging activity of the DPPH radical was observed at 67.34 mg/ml. These data suggest that rosemary extract may be suitable as a topical prophylactic or therapeutic agent for herpes viral infections. However, further research is required to elucidate the plant’s active constituents, which may be useful in drug development.

## Introduction

Herpes simplex viruses 1 and 2 (HSV-1 and HSV-2) belong to the herpesviridae family, primarily causing oral herpes lesions with HSV-1 and genital lesions with HSV-2 [1]. During the primary infection, HSV causes damage to the central nervous system, and can even result in encephalitis and meningitis [2]. Infections in immune-compromised patients are most severe and have been reported to be lethal [3]. Treatment of herpes infections is thus a major cause of concern owing to the difficulty in eliminating it from the ganglion, the high cost of treatment, and the increasing drug resistance.

At present, the standard therapy for the management of HSV infections is based on inhibition of the viral DNA polymerase by nucleoside analogs [4], including acyclovir, penciclovir, and their derivatives [4,5]. The resistance of HSV to acyclovir has become an important clinical problem, especially in immune-compromised patients undergoing long-term therapy [5]. Antiviral drugs may not be a perfect choice in many cases due to drug resistance [6]. Hence, it is essential to develop new antiviral agents, which can act against a broad range of viruses.

Rosemary (*Rosmarinus officinalis*, L.) is an herb commonly used as a spice and flavoring agent in food processing. The leaves are a very good source of carnosol, carnosic acid, rosmanol, 7-methyl-epirosmanol, isorosmanol, rosmadial, and caffeic acid, which have substantial *in vitro* antioxidant activities [7]. Antibacterial effects of rosemary have also been reported [8–10] and linked to their polyphenolic composition. Furthermore, rosemary oil may be used in drug-resistant infections [11]. Rosemary leaves possess a variety of biological activities, including anti-cancer and anti-inflammatory effects [12].

Traditional drugs have been used to prevent or treat HSV infections for many years. Presently, new antiviral agents with efficacy and without severe undesirable effects are being studied. Previous studies have suggested that some essential oils are effective against HSV-1 [13,14]. Therefore, the present work was undertaken to develop new anti-herpes drugs.

## Materials and methods

### Viruses and cell lines

Vero cells were grown in Dulbecco’s modified Eagle’s medium (DMEM; Gibco, Brazil) supplemented with 10% fetal bovine serum (FBS; Gibco, Brazil) and gentamycin (80 µg/ml). The cells were maintained at 37°C in a humidified atmosphere with 5% CO_2_. The herpes simplex virus types 1 and 2 were propagated in Vero cells and titrated based on plaque-forming units using plaque assay counts, as previously described [4], and stored at −80°C until the experiments were performed.

### Plant extractions

Dried rosemary (*R. officinalis*, L.) fine powder (100 g) was extracted in water at a 50× (w/v) concentration for 24 h at room temperature (30 ± 2°C). The extract was filtered, lyophilized, and stored at −20°C.

### Determination of flavonoid and phenol content

The aluminum chloride colorimetric method was used for total flavonoid content determination in the sample following the previously described method [15]. The total polyphenolic content was determined using a Folin-Ciocalteau reagent, which measures the oxidation of polyphenols to a blue-colored complex with a maximum absorbance at 750 nm [16].

### Determination of DPPH radical scavenging activity

The free radical scavenging capacity was evaluated by the 2,2-diphenyl-1-picrylhydrazyl (DPPH) assay [17], where the percent of DPPH decolorization of the sample was calculated by the following equation:

$$\text{DPPH (}\%\text{) scavenging activity = }\left(\frac{{\text{A}}_{\text{control}}-{\text{A}}_{\text{sample}}}{{\text{A}}_{\text{control}}}\right)\times 100$$

where *A*_control_ is the absorbance of DPPH solution without the sample and *A*_sample_ is the absorbance of DPPH solution with the sample. Superoxide anion scavenging activity was measured by determining the amount of reduced phenazine methosulfate [17]. Vitamin C was used as a positive control.

### Superoxide radical scavenging assay

This activity was estimated by the reduction of nitro blue tetrazolium (NBT) based on the previously described method [18]. The non-enzymatic phenazine methosulfate-nicotinamide adenine dinucleotide (PMS/NADH) system forms superoxide anion (O_2_·^−^) radicals, which reduce NBT to a purple formazan days. Briefly, to a one 1 ml reaction solution contained 20 mM phosphate buffer (pH 7.4), 73 μM NADH, 50 μM NBT, 15 μM PMS, and different concentrations (20–100 mg/ml) of extract solution. After incubation at room temperature for 5 min, the color intensity was measured at 562 nm against the blank to determine the amount of formed day. Vitamin C was used as positive control.

### Reducing power assay

The Fe^3+^ reducing power of the extract was determined according to a method by Abdel Moneim [18]. Briefly, 50, 100, 150, and 200 µl of the samples were mixed with 1.9 ml 0.2 M phosphate buffer, pH 6.6, and 2 ml of 1% potassium ferricyanide. The mixture was then incubated at 50°C for 20 min. Afterward, the mixture was stopped by adding 2 ml of 10% trichloroacetic acid and then centrifuging at 3000 rpm for 10 min. The upper layer of supernatant (2 ml) was mixed with distilled water (2 ml) and 0.1% FeCl_3_ solution (0.5 ml). The absorbance was measured at 700 nm against a blank with a spectrophotometer and vitamin C was used as a standard. Reaction mixtures with a higher absorbance indicated a greater reducing power.

### Cytotoxicity assay

Vero cells were exposed to different rosemary concentrations (1–5000 µg/ml) for 72 h and an MTT assay (3-(4,5-dimethylthiazol-2,5-diphenyl tetrazolium bromide) was performed to assess metabolic activity and therefore, cytotoxicity [4].

### Antiviral activity assay

The antiviral activity assay was performed as previously described by Schnitzler [14]. Acyclovir (Sigma®, St. Louis, MO, U.S.A.) at 0.05–2 μg/ml served as the positive control.

### Viral plaque number reduction assay

**A** previously described procedure was followed for this assay [4], with minor modifications. Approximately 100 plaque-forming units of HSV-1 and HSV-2 were adsorbed for 1 h at 37°C on confluent cells and overlaid with MEM plus 1.5% carboxymethyl cellulose (CMC, Sigma®, St. Louis, MO, U.S.A.), either in the presence or absence of different concentrations of orange peel. After 72 h, the cells were fixed and stained with naphthol blue black (Sigma) and plaques were counted. The 50% inhibitory concentration (IC_50_) was defined as the concentration that inhibits the formation of 50% of the viral plaques when compared with untreated controls [19].

### Statistical analysis

Data are presented as the mean ± the standard error of the mean (x̄ ± SEM) of at least three separate experiments.

## Results and discussion

**Figure 1:**
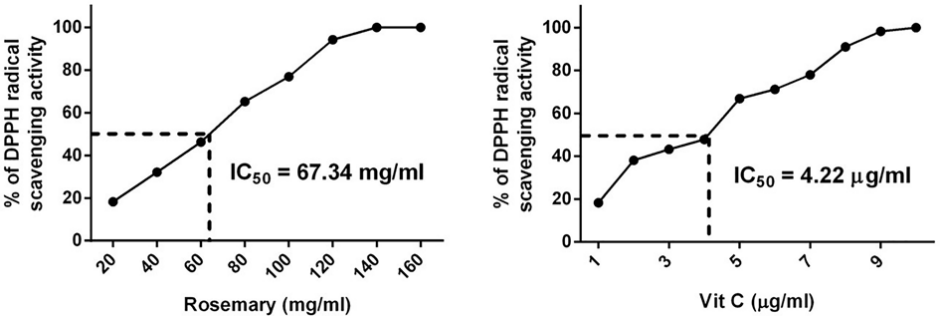
Determination of DPPH radical scavenging activity The antiradical activities of rosemary extract and ascorbic acid against the DPPH radical, as measured by changes in absorbance at 517 nm.

The total polyphenolic content in aqueous rosemary extract was 35.6 μg/mg gallic acid equivalents of polyphenols/mg extract, whereas the flavonoid content was 22.3 μg/mg quercetin equivalents of flavonoids/mg extract (Table 1). Similarly, Kim et al. [20] reported that a hot water extract of rosemary had 42.35 mg total phenolics/g and 26.98 μg/mg quercetin equivalents of flavonoids/mg extract. However, Couto et al. [21] obtained low polyphenol and flavonoid concentrations. The radical scavenging activity of the extract was 67.34 mg/ml, indicating that rosemary is a good antioxidant (Figure 1). The correlation between antioxidant activity and presence of polyphenols has been widely studied in herbs [20,22] and reported by Estevez et al. [23], who isolated carnosic acid from rosemary.

**Table 1:** Total phenolics and flavonoids contents of rosemary extracts

Conditions	Total phenolics^*^	Total flavonoids^†^
Rosemary extract	35.6 ± 0.8	22.3 ± 1.1

* Total phenolics are expressed as μg/mg gallic acid equivalent of polyphenols/mg extract.

† Flavonoids are expressed as μg/mg quercetin equivalents of flavonoids/mg extract.

Data are represented as mean ± SEM of three independent experiments each performed in duplicate.

**Figure 2:**
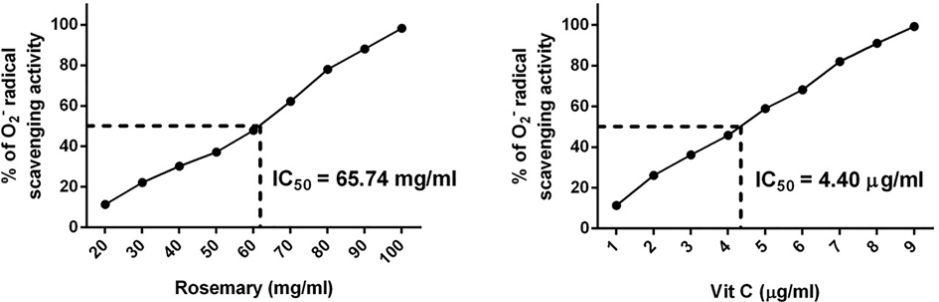
Determination of superoxide anion scavenging activity The antiradical activities of rosemary extract and ascorbic acid against O_2_·^−^ radicals, as measured by changes in absorbance at 560 nm.

Figure 2 shows the dose-response curves for the superoxide anion (O_2_·^−^) radical scavenging activity of the rosemary extract. The results indicate that the O_2_·^−^ radical scavenging activity was 65.74 mg/ml, suggesting that rosemary extract possesses strong antioxidant effects due to superoxide anion radical scavenging property.

Oxidation by NADPH oxidase leads to the generation of O_2_·^−^, which can be converted to H_2_O_2_ by superoxide dismutase (SOD) or reacts with nitric oxide (NO^·^) to form peroxynitrite. Hydrogen peroxide can be further converted to oxygen and water by catalase and glutathione peroxidase. Superoxide radicals have been observed to inactivate enzymes, degrade DNA, kill cells, and damage cell membranes [24]. The antioxidant activity of rosemary extract was stronger than the superoxide anion scavenging activity.

**Figure 3:**
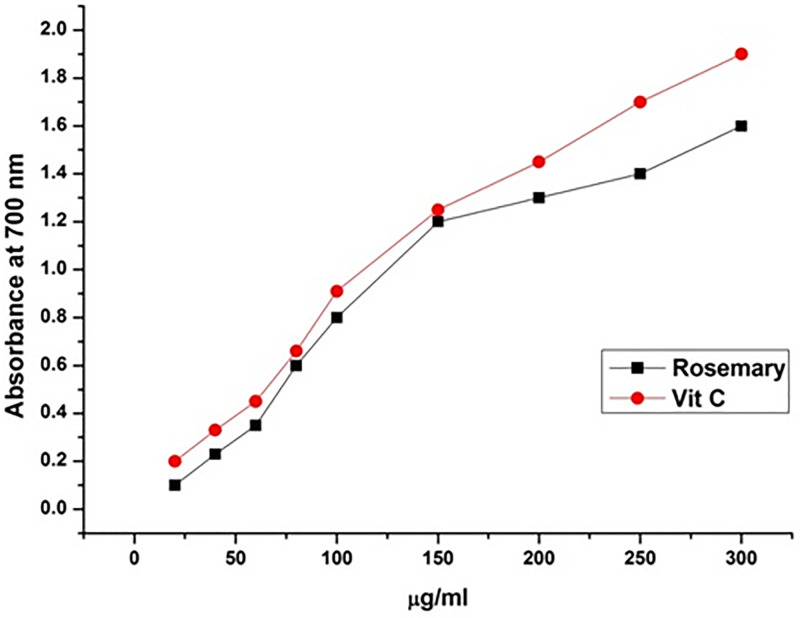
Reducing power of rosemary extract Reducing power of rosemary extract and ascorbic acid against Fe^3+^ ions, as measured by changes in absorbance at 700 nm.

The reducing power of a compound may serve as a significant indicator of its potential for antioxidant activity. During the reducing power assay, the presence of reductants (antioxidants) in the tested samples resulted in the reduction of the ferricyanide (Fe^3+^) complex to the ferrous form (Fe^2+^). Figure 3 shows that Fe^2+^ was monitored by measurement of absorbance at 700 nm, which increased linearly with increasing rosemary extract concentrations in the reaction mixture. It has been reported that a substances reducing power may likely be due to its hydrogen-donating ability [25].

**Figure 4:**
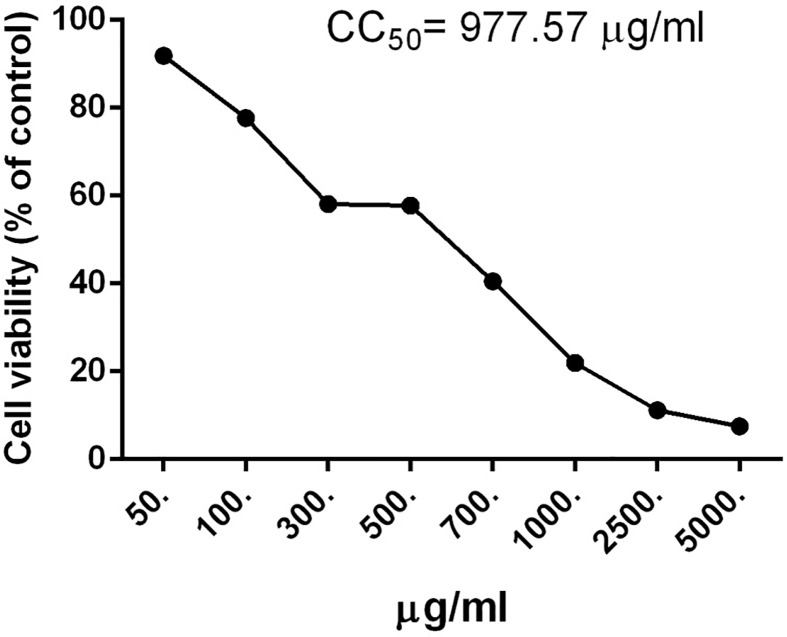
Cytotoxicity assay The cytotoxic effects of rosemary extract evaluated by MTT in Vero cells.

The MTT assay results (Figure 4) show that the rosemary extract cytotoxicity in cultured Vero cells was up to 977 µg/ml, indicating that rosemary extract had low toxic effects. This assay was previously used to discriminate between antiviral and cytotoxicity concentrations of various essential oils [26].

To study the antiviral activity of rosemary extract against HSV-1 and HSV-2, the cytopathic inhibitory assay was performed to determine the cytopathic effects (CPEs) of the viruses and the data are shown in Table 2. A confluent monolayer of Vero cells was infected with HSV-1 and HSV-2 viruses. A high concentration (50 µg/ml) of rosemary extract showed moderate antiviral activity against the viruses, with a CPE seen at 35 µg/ml.

**Table 2:** The antiviral activity of rosemary extract against HSV-1 and HSV-2

Rosemary	Cell cytopathic effect	
	HSV-1	HSV-2
10 µg/ml	+	+
15 µg/ml	+	+
20 µg/ml	+	+
25 µg/ml	+	+
30 µg/ml	−	+
40 µg/ml	−	+
50 µg/ml	−	−

**Figure 5:**
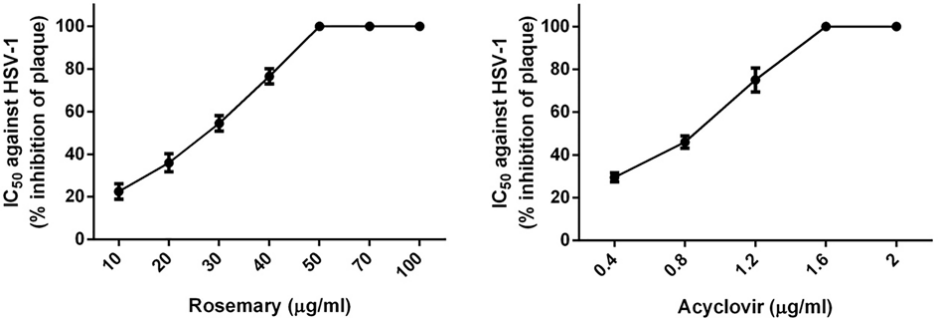
The anti-herpes virus 1 activity of rosemary extract and acyclovir as a standard antiviral agent *In vitro* activity of rosemary extract and acyclovir against anti-HSV-1. IC_50_ is the concentration where a 50% cytotoxic effect is observed. Data are represented as mean ± SEM of two independent experiments, each performed in triplicate.

As shown in Figure 5, rosemary extract at 30 µg/ml caused 55% inhibition of HSV-1 plaques and 100% inhibition against HSV-1 at 50 µg/ml. The 50% inhabitation of acyclovir against HSV-1 was less than 0.9 µg/ml. A plaque inhibition assay was conducted to determine the IC_50_.

**Figure 6:**
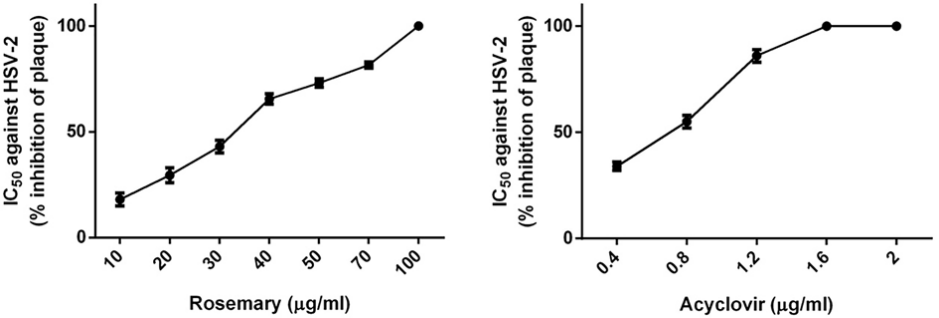
The anti-herpes virus 2 activity of rosemary extract and acyclovir as a standard antiviral agent *In vitro* activity of rosemary extract and acyclovir against anti-HSV-2. IC_50_ is the concentration where a 50% cytotoxic effect is observed. Data are represented as mean ± SEM of two independent experiments, each performed in triplicate.

Figure 6 shows the *in vitro* activity of rosemary extract against anti-HSV-2. The HSV-2 strain was more sensitive, with 40 µg/ml of rosemary extract causing 65% inhibition of HSV-2 plaques and showing 100% inhibition at 50 µg/ml. The 50% inhabitation of acyclovir against HSV-2 was less than 0.8 µg/ml. The anti-HSV-1 and 2 effects may be due to enhanced absorption of the extracts into the Vero cells. It may be that a phytochemical is participating in phenolic binding with the protein coat of the virus, blocking ligands from the viral surface, thus inactivating the virus. However, further studies are needed to confirm these observations.

Several studies showed that polyphenols, triterpenes, anthraquinones, saponins, and polysaccharides that were isolated from natural plants can inhibit the replication of herpes viruses [27]. A large number of plant-derived and synthetic anti-HSV agents have also been described [4,28].

## Conclusions

The present *in vitro* studies revealed that rosemary extract exhibited antiviral activity against HSV-1 and HSV-2, along with antioxidant effects. Further studies are necessary to identify the mechanism responsible for this activity.

## Data Availability

All data generated or analyzed during this study are included in this article.

## Abbreviations

CPE: cytopathic effect
DPPH: 2,2-diphenyl-1-picrylhydrazyl
HSV: herpes simplex virus
NADH: nicotinamide adenine dinucleotide
NBT: nitro blue tetrazolium
PMS: phenazine methosulfate
